# Hollow Mesoporous Fe_2_O_3_ Nanospindles/CNTs Composite: An Efficient Catalyst for High-Performance Li-O_2_ Batteries

**DOI:** 10.3389/fchem.2019.00511

**Published:** 2019-07-25

**Authors:** Hairong Xue, Yiou Ma, Tao Wang, Hao Gong, Bin Gao, Xiaoli Fan, Juanjuan Yan, Xianguang Meng, Songtao Zhang, Jianping He

**Affiliations:** ^1^Jiangsu Key Laboratory of Materials and Technology for Energy Conversion, College of Materials Science and Technology, Nanjing University of Aeronautics and Astronautics, Nanjing, China; ^2^Photofunctional Materials Research Platform, College of Materials Science and Engineering, North China University of Science and Technology, Tangshan, China; ^3^Testing Center, Yangzhou University, Yangzhou, China

**Keywords:** hollow mesoporous structure, carbon support, transition metal oxides, cathodic catalyst, Li-O_2_ batteries

## Abstract

The design of mesoporous or hollow transition metal oxide/carbon hybrid catalysts is very important for rechargeable Li-O_2_ batteries. Here, spindle-like Fe_2_O_3_ with hollow mesoporous structure on CNTs backbones (Fe_2_O_3_-HMNS@CNT) are prepared by a facile hydrolysis process combined with low temperature calcination. Within this hybrid structure, the hollow interior and mesoporous shell of the Fe_2_O_3_ nanospindles provide high specific surface area and abundant catalytical active sites, which is also beneficial to facilitating the electrolyte infiltration and oxygen diffusion. Furthermore, the crisscrossed CNTs form a three-dimensional (3D) conductive network to accelerate and stabilize the electron transport, which leads to the decreasing internal resistance of electrode. As a cathodic catalyst for Li-O_2_ batteries, the Fe_2_O_3_-HMNS@CNT composite exhibits high specific capacity and excellent cycling stability (more than 100 cycles).

## Introduction

To meet the global energy demand, the development of the clean and sustainable energy storage or conversion devices is very important (Tarascon and Armand, 2011; Lu et al., 2014; Wang et al., 2017; Zhang et al., 2018; Gao et al., 2019). Rechargeable Li-O_2_ battery has attracted wide attention as a new energy storage device, due to its high theoretical energy density (~3,500 Wh kg^−1^) (Tarascon and Armand, 2011; Lu et al., 2014). However, the practical application of Li-O_2_ batteries still suffer a series of problems, including high overpotentials, low rate capacity and poor cycle stability, which primarily originates from its sluggish kinetics for oxygen reduction reaction (ORR) and oxygen evolution reaction (OER) (Bruce et al., 2012; Wang et al., 2014). At the cathode (air electrode), the gradual formation of the insoluble discharge products Li_2_O_2_ may block the inward oxygen diffusion of and electrolyte infiltration, which results in the rapid decline of battery performance (Zhao et al., 2015; Wang et al., 2016). To overcome these challenges, the design and development of the high-performance catalysts for oxygen-involved reactions are highly desired for the Li-O_2_ batteries.

In recent years, a lot of the efforts have been devoted to investigate the highly active and stable catalysts for the Li-O_2_ batteries, such as carbons, precious metals, and transition metal oxides. As the common catalysts, the inexpensive carbon materials have high surface area, nevertheless, their limited catalytic activity for both OER and ORR restricts the battery performance of the Li-O_2_ batteries (Girishkumar et al., 2010; Shui et al., 2013). Since the nanoporous gold (NPG) was used as a cathode catalyst, various precious metals (e.g., Ru, RuO, and Pd) have been adopted in the Li-O_2_ batteries (Peng et al., 2012; Lu et al., 2013; Ottakam Thotiyl et al., 2013; Li et al., 2014, 2015). Although the cyclic stability can be distinctly enhanced by precious metal catalysts, the battery capacity is severely restricted because of the highly chemical formula weight. Moreover, high price of precious metals also hinders the large-scale commercialization in the Li-O_2_ batteries. Benefiting from the low cost, high stability and good catalytic performance, transition metal oxides have been proposed as the promising catalysts for the Li-O_2_ batteries (Wang H. et al., 2012; Chen et al., 2016; Gong et al., 2016, 2018a,b; Xue et al., 2016a,c; Dai et al., 2017; Tan et al., 2017; Feng et al., 2019). Many researches have indicated that Fe-based materials possess high catalytic activities for ORR in fuel cells and OER in water electrolysis (Bates et al., 2016; Song et al., 2019). Recently, some reports began to focus on iron oxides (Fe_2_O_3_), which can serve as the cathode catalyst in Li-O_2_ batteries (Zhang et al., 2014). These works show the enhanced electrochemical performance (e.g., higher capacity and lower overpotentials) of the Li-O_2_ batteries, however the cycling performance still needs to further improve. Therefore, it is necessary to explore an effective approach to enhance the catalytic performance of Fe_2_O_3_-based materials.

Tailoring of the morphology is an important method for obtaining the high-performance catalysts in various electrochemical application. Mesoporous hollow architectures show high surface area and large pore volume, which offers fast electron transfer paths and facilitates the electrolyte infiltration (Kresge et al., 1992; Inagaki et al., 2002; Malgras et al., 2016). Normally, mesoporous or hollow metallic oxide are prepared through the template-based methods, using either hard templates (e.g., carbon sphere and mesoporous silica) or soft templates (e.g., surfactants and polymer) (Attard et al., 1997; Crossland et al., 2013; Liu et al., 2015; Xue et al., 2016d). For these template methods, the multi-step processes are unavoidable, and the mesoporous of hollow structures may be damaged after removing the templates. Moreover, the resultant mesoporous or hollow frameworks often show poor crystalline degree and even amorphous, which limits their electrochemical performance (Lin et al., 2015). It should be noted that the low electronic conductivity is an intrinsical characteristic of the most metallic oxides. Although most of pure carbon materials possess low catalytic activity for OER and ORR, they are identified as the good catalyst support due to their high electric conductivity and large specific surface area (Hsin et al., 2007; Stein et al., 2009; Wu et al., 2009; Xia et al., 2018). Among various carbon materials, the carbon nanotubes (CNTs) show low density, very high strength, high chemical stability, and excellent conductivity (Sathiya et al., 2011; Wang Z. et al., 2012; Ma et al., 2018). These advantages are beneficial to fabricate hybrid or composite materials in many applications by using CNTs as useful substrates. However, the fabrication of mesoporous hollow Fe_2_O_3_ with high crystalline degree supported on CNTs by a simple and effective method is still an important challenge.

Inspired by the above idea, we proposed a Fe_2_O_3_/CNTs composite (denoted as Fe_2_O_3_-HMNS@CNT) prepared by using a simple hydrolysis reaction combined with heat treatment, in which spindle-like Fe_2_O_3_ with hollow mesoporous structure grown on CNTs backbones. Their hollow interior and mesoporous shell with high specific surface area offer abundant catalytical active sites for OER and ORR, which also promotes the diffusion and infiltration of electrolyte. Furthermore, a great deal of the crisscrossed CNTs form the three-dimensional (3D) conductive network, which benefits the fast and stable electron transport. As a cathodic catalyst for Li-O_2_ batteries, the Fe_2_O_3_-HMNS@CNT exhibits good battery performance, especially excellent outstanding cycling stability (100 cycles).

## Experimental Methods

### Hollow Mesoporous Fe_2_O_3_ Nanospindles on CNT Backbones

Carbon nanotubes (CNTs, 30–60 nm in diameter and 5–15 μm in length) were purchased from Shenzhen Nanotech Port Co. Ltd (Shenzhen, China). CNTs were refluxed in HNO_3_ and then rinsed with distilled water. After drying, the obtained CNTs (10 mg) was dispersed in FeCl_3_ solution (20 mL, 0.12 M) under sonication for 1 h. Then, the suspension was heated at 75°C for 6 h in an oil bath with stirring. After several rinsing combined with sonication, the products were dried at 60°C. Finally, the above products were annealed at 400°C for 4 h in air by using a slow heating rate (0.5°C min^−1^) to form hollow mesoporous Fe_2_O_3_ nanospindles on CNT backbones.

### Materials Characterization

The X-ray diffraction (XRD, Bruker D8 advance) with Cu Ka radiation (λ = 1.5406 Å) is used to analyze the crystal structure of the sample. The N_2_ adsorption–desorption measurements conducted on a ASAP-2010 analyzer to investigate the pore structure. The Brunauer-Emmet-Teller (BET) method is used to calculate the specific surface area. The morphology and microstructure are observed by using field-emission scanning electron microscope (FE-SEM, Hitachi S-4800) and high-resolution transmission electron microscope (HR-TEM, JEOL JEM-2100), respectively.

### Electrochemical Measurement

The electrochemical measurements are tested under two-electrode system at room temperature in the pure O_2_ atmosphere, using Li plate as the counter and reference electrode. The work electrode is prepared by mixing Fe_2_O_3_-HMNS@CNT catalyst (50 wt %), Super P (45 wt %) and polytetrafluoroethylene (PTFE) binder (5 wt %), followed by vacuum drying at 100°C for 12 h. The capacity and current density of the sample is calculated by the whole electrode' weight. For the assembling of Li-O_2_ battery, the working electrode and Li plate are separated by a glass-fiber separator in coin cell. The tetraethylene glycol dimethyl ether (TEGDME) containing lithium bis(tri-fluoromethane-sulfonyl)imide (LiTFSI) (1 M) is used as electrolyte. The Land 2100 Charge/Discharge instruments are carried to test the electrochemical measurements.

## Results and Discussion

**Figure 1:**
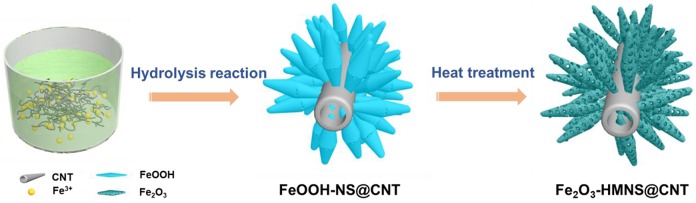
Schematic illustration of the preparation of the Fe_2_O_3_-HMNS@CNT composite.

Figure 1 illustrates the fabrication of the mesoporous hollow Fe_2_O_3_ nanocrystals covered on CNT by a simple hydrolysis reaction combined with heat treatment. In order to facilitate the nucleation and anchoring of the nanocrystals on the CNT surface, these CNTs are functionally modified with carboxylic or hydroxyl groups under acidic reflux. During the subsequent stirring process, the electropositive Fe^3+^ ions can preferential adsorb the electronegative oxygen-containing groups on CNTs by the electrostatic attraction. Due to the hydrolysis of Fe^3+^ ions followed by the olation/oxolation of the FeO_6_ units, the formed spindle-like β-FeOOH nanocrystals can be spontaneously grown on CNT backbones, which avoids the addition of any structure-directing agent. The TG/DSC is used to explore the reactions in the final annealing step (Figure S1). The TG curve show two mainly weight loss processes at 300°C and 300~400°C during the annealing step, which are attributed to the conversion from FeOOH into Fe_2_O_3_. The slowly intramolecular dehydration results in the slow weight loss before 300°C. After 300°C, a distinct weight loss can be found, which is originated from the fast removal of the H_2_O molecules. After annealing treatment in air, the spindle-like β-FeOOH nanocrystals are converted to hollow mesoporous α-Fe_2_O_3_ nanospindles on CNT backbones (denoted as Fe_2_O_3_-HMNS@CNT) by the thermal dehydroxylation together with the lattice shrinkage.

**Figure 2:**
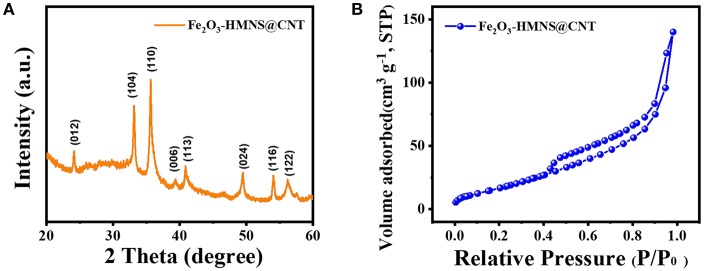
**(A)** XRD patterns, **(B)** N_2_ adsorption–desorption isotherms of the Fe_2_O_3_-HMNS@CNT composite.

The crystalline structure of the Fe_2_O_3_-HMNS@CNT composite is confirmed by XRD analysis. As shown in Figure 2A, the XRD pattern exhibits several intensively diffraction peaks at the 2θ values of about 24.2, 33.2, 35.6, 39.3, and 40.9°, which are indexed to the (012), (104), (110), (006), and (113) facets of the hexagonal α-Fe_2_O_3_. This result of XRD is in accord with the standard card of α-Fe_2_O_3_ (JCPDS no. 33-0664). The pore structure of the Fe_2_O_3_-HMNS@CNT composite is analyzed by N_2_ adsorption–desorption isotherms (Figure 2B). It can be seen that there is a type-IV curve of the Fe_2_O_3_-HMNS@CNT with a distinctly hysteresis loop on N_2_ adsorption–desorption isotherms, which indicates the typically mesoporous structure (Deng et al., 2007; Xue et al., 2016b). Moreover, the nitrogen uptake is found from 0.40 to 0.70 (P/P_o_), which can be attributed to the mesoporous materials' capillary condensation of nitrogen. Based on N_2_ adsorption–desorption isotherms, the specific surface area is calculated to be 97 m^2^ g^−1^. The above results indicate that the Fe_2_O_3_-HMNS@CNT has a typically mesoporous structure with high specific surface area.

**Figure 3:**
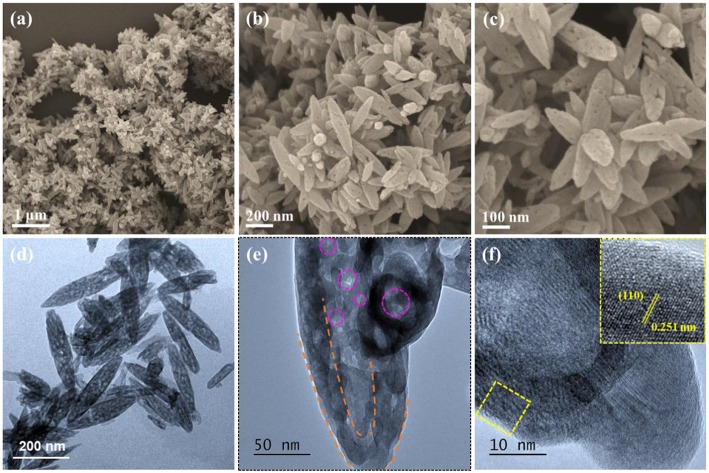
**(a–c)** FESEM, **(d–f)** HRTEM images of the Fe_2_O_3_-HMNS@CNT composite.

The morphologies and structures of the Fe_2_O_3_-HMNS@CNT composite are observed by scanning electron microscopy (SEM) and transmission electron microscopy (TEM). As shown in Figure S2, the CNT shows the typical one-dimensional tubular structure with a diameter of ~40 nm. Figure 3a reveals a panoramic view of the sample, in which the spindle-like nanocrystals grown on the entire surface of the sinuous CNTs backbones. It can be noted that there are many obvious porous within the Fe_2_O_3_ nanospindles, as shown in Figures 3b,c. TEM observation is used to further investigate the internal structure of the sample. The spindle-like Fe_2_O_3_ nanocrystals have a diameter of around 80 nm and a length of about 250 nm (Figure 3d), which show a well-developed hollow interior (marked by orange dotted line) and a typically mesoporous shell (marked by purple dotted line) (Figure 3e). During the calcining process, the decomposition-oxidation of the FeOOH can release abundant H_2_O and gases, which leads to the formation of mesoporous structure. On the other hand, the density of FeOOH (3 g cm^−3^) is lower than that of the hematite (Fe_2_O_3_, 5.3 g cm^−3^), so some internal mesoporous slowly forms the larger porous to maintain the spindle-like structure during the lattice shrinkage process, thus leading to the formation of the hollow interior together with the mesoporous shell. As shown in Figure 3f, some clear lattice fringes with an interplanar spacing of 0.251 nm in a single Fe_2_O_3_ nanospindle, corresponding to the (110) planes of the hexagonal α-Fe_2_O_3_, which confirms the formation of the α-Fe_2_O_3_ with high crystallinity.

**Figure 4:**
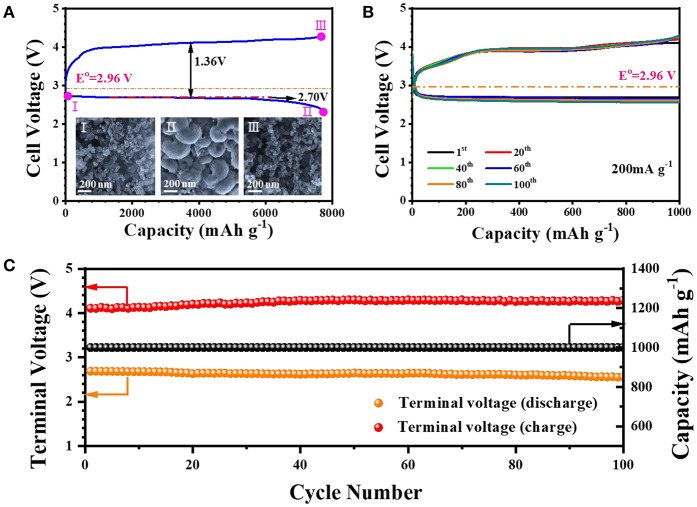
**(A)** The fully discharging and charging curves in the first cycle and the SEM images [inset of **(A)**], **(B)** cyclic stability tested under a limited capacity (1,000 mAh g^−1^), and **(C)** the variation of the terminal charge/discharge voltages and specific capacity over 100 cycles of the Fe_2_O_3_-HMNS@CNT-based cathode.

Inspired by the superiorities of material and structure, the Fe_2_O_3_-HMNS@CNT composite is investigated as the cathodic catalyst for rechargeable Li-O_2_ batteries. Figure 4A shows the discharge and charge curves of the sample, which is tested under O_2_ atmosphere with a current density of 200 mA g^−1^. The Fe_2_O_3_-HMNS@CNT-based cathode exhibits a large discharge capacity of 7,730 mAh g^−1^ and a high discharge plateau (~2.70 V) with the low overpotential for discharging (0.26 V). Owing to the low overpotential (1.10 V) for the charge process, the coulombic efficiency of the sample is closed to 100%. The reversible formation/decomposition of the discharging product Li_2_O_2_ can be confirmed by SEM (inset of Figure 4A). After fully discharging (defined as II), it is seen that a mass of the Li_2_O_2_ particles with the typically toroid-like shape uniformly cover on the electrode surface, which is observably different from the fresh electrode (defined as I). When the electrode is fully charged, the formed Li_2_O_2_ particles is rarely observed (defined as III), indicating high reversibility of the Fe_2_O_3_-HMNS@CNT-based cathode. We further evaluate the cycling performance of the Fe_2_O_3_-HMNS@CNT-based cathode, tesed under the limited capacity (1,000 mAh g^−1^). It can be found that the sample has the small discharge and charge overpotentials of 0.24 and 0.93 V in the first cycle, respectively, which implies the high catalytic activity for both OER and ORR (Figure 4B). After 100 cycles, no distinct change of the specific capacities of the sample is observed in discharging/charging curves. Moreover, the discharging and charging terminal voltages can still maintain in 2.6 and 4.3 V. These results exhibit the excellent cycling stability of the Fe_2_O_3_-HMNS@CNT-based cathode (Figure 4C). As a reference, the cycling stability of the Fe_2_O_3_-HMNS@CNT-based cathode is much better than those of previously reported non-precious metal/metal oxide-based materials (Table S1).

As the cathodic catalyst for rechargeable Li-O_2_ batteries, the as-prepared Fe_2_O_3_-HMNS@CNT composite exhibits the excellent cycling stability, which not only benefits by the intrinsic material characteristics but also dependents on the morphology and structure. On the one hand, these largely separated Fe_2_O_3_ nanospindles directly grown on the CNT backbones, which makes them accessible to the electrolyte. Different from the conventional nanoparticle catalysts, the adverse agglomeration of the Fe_2_O_3_-HMNS@CNT can be effectively decreased, avoiding the elimination of the active interfaces. The Fe_2_O_3_ with a hollow interior and a mesoporous shell offers high specific surface area and a mass of catalytical active sites, which also facilitates the diffusion and infiltration of electrolyte. On the other hand, the 3D conductive network composed of the crisscrossed CNTs ensures the fast and stable electron transport, leading to the lower internal resistance of electrode.

## Conclusion

In summary, we successfully fabricated hollow mesoporous Fe_2_O_3_ nanospindles on CNTs (Fe_2_O_3_-HMNS@CNT) though a simple hydrolysis reaction followed by a heat treatment, which are served as cathodic catalyst for Li-O_2_ batteries. In this catalyst design concept, the spindle-like Fe_2_O_3_ nanocrystals possess the hollow interior and mesoporous shell, which not only provides high specific surface area and abundant catalytical active sites but also facilitates the diffusion and infiltration of electrolyte. Moreover, the 3D conductive network formed by the crisscrossed CNTs ensures the fast and stable electron transport, reducing internal resistance of electrode. Benefiting from the intrinsic material characteristics and structural superiorities, the Fe_2_O_3_-HMNS@CNT catalyst shows high specific capacity and excellent cyclic stability.

## Data Availability

All datasets generated for this study are included in the manuscript/Supplementary Files.

## Author Contributions

HX, TW, and JH conceived this research work. HX performed the experiments and wrote the manuscript. YM, HX, HG, BG, XF, and JY tested the electrochemical performance. XM and SZ contributed to analyze the experimental results. All authors read and approved the final manuscript.

### Conflict of Interest Statement

The authors declare that the research was conducted in the absence of any commercial or financial relationships that could be construed as a potential conflict of interest.
