# Supplementary material for: Hollow Mesoporous Fe2O3 Nanospindles/CNTs Composite: An Efficient Catalyst for High-Performance Li-O2 Batteries
Source: Front Chem. 2019 Jul 25;7:511. doi: 10.3389/fchem.2019.00511 (PMC6672713; doi:10.3389/fchem.2019.00511)
Supplement: Supplementary file 1 [file Data_Sheet_1.pdf]

Supplementary Material

Supplementary Figures

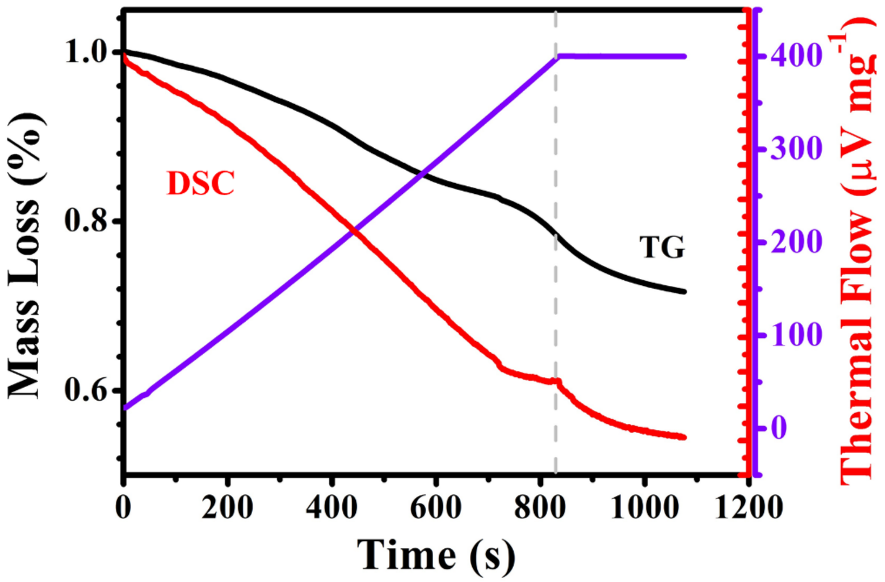

Figure S1. The TG/DSC curves of the FeOOH@CNT precursor simulated the annealing process under air atmosphere.

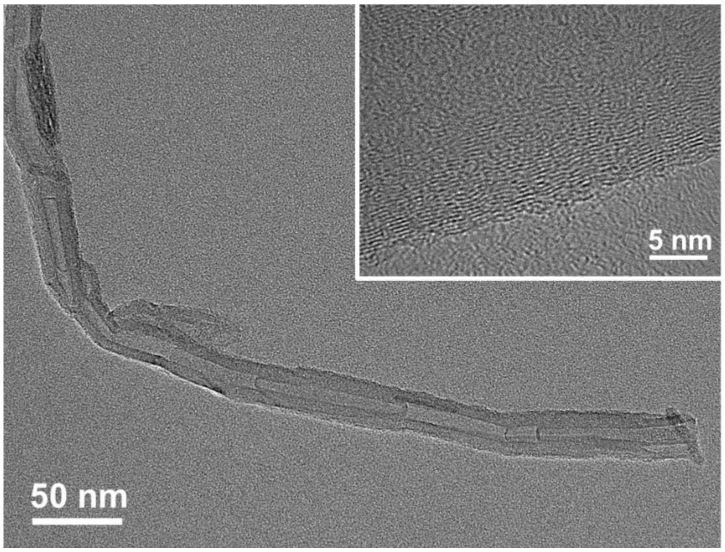

Figure S2. The TEM and HRTEM (inset) of images of CNT.

Supplementary Tables

Table S1. comparisons of electrochemical performance of the Fe_2O_3-HMNS@CNT with the recently reported non-precious metal/metal oxide-based materials for Li-O_2 batteries.

Referance	Type of material	Current density	Limited capacity	Cycle number
**This work**	**Fe_2O_3-HMNS@CNT**	**200 mA g^{-1}**	**1000 mAh g^{-1}**	**100**
1	TiCrOx	50 mA g^{-1}	200 mAh g^{-1}	20
2	CNT	200 mA g^{-1}	1000 mAh g^{-1}	50
3	Holey Graphene	0.2 mA cm^{-2}	2 mAh cm^{-2}	20
4	α-MnO_2	200 mA g^{-1}	1000 mAh g^{-1}	70
5	3D Foam-Like $NiCo_2O_4$	200 mA g^{-1}	1000 mAh g^{-1}	30
6	Co_3O_4/ Ni foam	200 mA g^{-1}	500 mAh g^{-1}	80
7	graphitic porous carbon-Co_3O_4	250 mA g^{-1}	500 mAh g^{-1}	50
8	Co_3O_4 nanotube	25 μA cm^{-2}	1000 mAh g^{-1}	40

References:

1. Lai, N., Cong, G., Liang, Z., and Lu, Y. (2018) A Highly Active Oxygen Evolution Catalyst for Lithium-Oxygen Batteries Enabled by High Surface-Energy Facets. *Joule* 2, 1511-1521.
2. Xu, S., Yao,Y., Guo, Y., Zeng, X., Lacey, S., Song, H. et al. (2018) Textile Inspired Lithium-Oxygen Battery Cathode with Decoupled Oxygen and Electrolyte Pathways. *Adv. Mater.* 30, 1704907
3. Lin, Y., Moitoso, B., Martinez-Martinez, C., Walsh, E., Lacey, S., Kim, J., et al. (2017) Connell, Ultrahigh-Capacity Lithium-Oxygen Batteries Enabled by Dry-Pressed Holey Graphene Air Cathodes. *Nano Lett.* 17, 3252-3260.
4. Gu, T., Agyeman, D., Shin, S., Jin, X., Lee, J., Kim, H., et al. (2018) α-MnO_2 Nanowire-Anchored Highly Oxidized Cluster as a Catalyst for Li-O_2 Batteries: Superior Electrocatalytic Activity and High Functionality, *Angew.Chem. Int. Edit.* 57, 15984-15989.
5. Liu, L., Wang, J., Hou, Y., Chen, J., Liu, H., Wang, J., et al. (2016) Self-Assembled 3D Foam-Like $NiCo_2O_4$ as Efficient Catalyst for Lithium-Oxygen Batteries. *Small* 12, 602-611.
6. Wu, F., Zhang, X., Zhao, T., Chen, R., Ye, Y., Xie, M., et al. (2015) Hierarchical mesoporous/macroporous Co_3O_4 ultrathin nanosheets as free-standing catalysts for rechargeable lithium-oxygen batteries. *J. Mater. Chem. A* 3, 17620-17626.
7. Tang, J., Wu, S., Wang, T., Gong, H., Zhang, H., Alshehri, S., et al. (2016) Cage-Type Highly Graphitic Porous Carbon-Co_3O_4 P olyhedron as the Cathode of Lithium-Oxygen Batteries. *ACS Appl. Mater. Interfaces* 8, 2796−2804.
8. Liu, L., Guo, H., Hou, Y., Wang, J., Fu, L., Chen, J., et al. (2017) A 3D hierarchical porous Co_3O_4 nanotube network as an efficient cathode for rechargeable lithium-oxygen batteries. *J. Mater. Chem. A* 5, 14673-14681.
